# Influence of the use of remineralizing agents on the tensile bond strength of orthodontic brackets

**DOI:** 10.1038/s41598-023-27390-0

**Published:** 2023-01-10

**Authors:** Milda Domantaitė, Giedrė Trakinienė

**Affiliations:** Department of Orthodontics, Lithuanian University of Health Sciences, Medical Academy, Lukšos –Daumanto 6, Kaunas, Lithuania

**Keywords:** Orthodontics, Fixed appliances, Fluoride varnish

## Abstract

This study aimed to evaluate the influence of the use of remineralizing agents on the tensile bond strength. The study sample consisted of 80 recently extracted molars, which were randomly divided into four experimental groups (n = 20): groups FG1 and FG30, in which fluoride varnish was used, and groups RG1 and RG30, in which Recaldent tooth mousse was used. The mesiobuccal surfaces served as experimental, and distobuccal as control (n = 80). Brackets were bonded to both surfaces and were submitted to a tension test at different time intervals (T1 and T30). Tensile bond strength (TBS) and the amount of adhesive remnant (ARI) were assessed. A statistically significantly lower mean of TBS compared to the control group was found only in the FG1 group (*p* < 0.001), and no significant difference was found between the other groups. The FG1 group showed significantly higher ARI scores (*p* < 0.001) compared to the control group. No significant difference was found between the other groups. In conclusion, bonding brackets one day after applying fluoride varnish significantly reduced the TBS, but after 30 days it was set back to an optimal value. The use of Recaldent before orthodontic treatment had no adverse effect.

## Introduction

Dental caries is one of the most common diseases in modern society, and the prevalence of it reaches about 44% of the world's population^1^. Despite considerable efforts by both dentists and other health professionals to reduce the incidence of caries, the prevalence has decreased by only 4% in the last thirty years^2^. For this reason, there is now an increasing focus on the prevention of tooth decay, which includes not only dietary correction, personal oral hygiene or the use of prebiotics and probiotics^1^, but also the use of substances such as fluoride and calcium phosphopeptide—amorphous calcium phosphate (CPP-ACP), a milk-derived protein which is gaining its popularity these days^3^. These substances can be applied professionally or used by the patients themselves^4, 5^.

This CPP-ACP delivers bioavailable calcium and phosphate ions and acts as a reservoir for these ions^6^. Acids release calcium and phosphate ions, which saturate the saliva and then deposit as a calcium phosphate compound on the exposed surface of the tooth^7^. CPP-ACP is thought to contribute to deeper ion penetration and thus remineralize not only the superficial enamel layer but also the deeper layers of hard tissue of the tooth, which may also improve the aesthetic appearance^8, 9^. This complex has antimicrobial properties in part because, by bounding to the dental pellicle, it inhibits the adhesion of *Streptococcus mutants*, highly cariogenic bacteria*,* to the tooth surface^10, 11^.

It is common knowledge that fluoride therapy reduces enamel demineralization and increases its resistance to organic acids^12^. Fluoride has a facilitating effect on the diffusion of calcium and phosphate ions into the demineralized surface. Thus, it can restore the crystalline enamel structure composed of fluorinated hydroxyapatites and fluorapatites, which are more resistant to acids than the primary crystals^13^.

It is worth noting that dental and occlusal anomalies are also common today. The prevalence varies from 29.2 to 93% of the world’s population^14, 15^, and the figure is as high as 71% among Europeans^16^. Due to the high prevalence of such anomalies and the improving economic conditions in both developing and developed countries, people are increasingly focusing on the aesthetics of a smile, leading to an increasing number of patients seeking beautiful smiles through orthodontic treatment^17^.

Fixed orthodontic appliances—braces—are most often used for the treatment of dental and occlusal anomalies. It is suggested that a minimum tensile bonding strength of 5.9–8 MPa between orthodontic brackets and teeth would be adequate for clinical orthodontic tooth movement^18^. The tensile bond strength of the brackets depends on many factors, such as the structure of the tooth, the etching time and the acid used, the type of bracket system, the type of bracket, or even previously done procedures on the teeth, such as teeth whitening with 35% hydrogen peroxide^19–22^. There is a significant influence on the structure of tooth enamel, as studies have shown that demineralized or fluorosis-damaged enamel surface significantly reduces the adhesion of the brackets^20, 23^. The research also indicates that fluoride agents reduce adhesive adherence^24^, but the data on the effect of remineralizing agents on tensile bond strength is controversial.

The study aimed to evaluate the influence of using remineralizing agents on the tensile bond strength.

## Methods

The present in vitro study was performed at the Department of Orthodontics and the Laboratory of Mechanical Engineering Faculty. Bioethical approval was obtained from the Lithuanian University of Health Sciences Bioethical Committee (No: BEC-OF-106) and the methods were carried out in accordance with the relevant guidelines. Informed consent was obtained from all subjects who participated in the study.

The power analysis with G*Power (Version 3.1.9.2)^25^ statistical software was used to determine the sample size. The parameters adopted were as follows: significance level of 5%, power test of 80%, the standard deviation of 3, and smallest effect of interest of 2. The calculation of the sample size was based on the following formula:

$$n = \frac{{\sigma^{2} \left( {Z\left( {\frac{\alpha }{2}} \right) + Z\left( \beta \right)} \right)^{2} }}{{\Delta^{2} }}$$

where n—the minimum sample size for each sample; Z(α/2) = 1.96 and Z(β) = 0.84 if α = 0.05 and β = 0.2; σ—standard deviation; Δ—the smallest clinically important difference.

$$n = \frac{{3^{2} (1,96 + 0,8416)^{2} }}{{2^{2} }} = 17,66$$

The sample size calculation showed that at least 18 specimens were needed in each group.

Over a period of 1 month, 80 extracted unhealthy and incurable human molars were collected, remnants of blood and soft tissues were removed, and the teeth were washed under a stream of distillated water. Only the molars with intact enamel surface were used for the study: no decay, no restorations, no cracks from the tooth extraction forceps and no hypoplastic areas. Before the study, the teeth were kept in saline, changing it daily.

The teeth were randomly divided into 4 experimental groups (n = 20): two groups (hereinafter referred to as Fluoride Groups *FG1* and *FG30)*, which used chemically cured fluoride varnish (Bifluoride 10, VOCO GmbH, Cuxhaven, Germany), and another two groups (hereinafter referred to as Recaldent Groups *RG1* and *RG30*), which used tooth mousse with bioactive calcium and phosphates (GC Tooth Mousse, GC Europe, Leuven, Belgium). In groups FG1 and RG1, brackets were bonded 1 day after the application of remineralizing agents, and in groups FG30 and RG30—30 days after application. The buccal surface of each tooth was divided into experimental (mesiobuccal) and control (distobuccal) (n = 80). As a result, 160 surfaces were used for the investigation. The brackets of the control group (distobuccal surfaces) were bonded and submitted to a tension test before applying the remineralizing agents to avoid contamination by them.

Before starting testing tensile bond strength, the buccal surface of each tooth was evaluated with a stereomicroscope (Stemi 2000-CS, Zeiss, Oberkochen, Germany) to assess the enamel cracks. The adhesive remnant index (ARI) was also evaluated after the brackets were debonded.

Remineralizing agents were applied according to the manufacturer's instructions. The proximal buccal surfaces of each group (experimental group) were polished with a rubber cup and non-fluoridated pumice, rinsed with distillated water and dried with a stream of air. The experimental surface (mesiobuccal) of the Recaldent tooth mousse groups (RG1 and RG30) was coated with Recaldent tooth mousse (GC Tooth Mousse, GC Europe, Leuven, Belgium) using a micro brush and left for 5 min according to the manufacturer's instructions, then rinsed with distilled water. The procedure was repeated every 6 h within the period of 5 days. Fluoride varnish (Bifluorid 10, VOCO GmbH, Cuxhaven, Germany) was applied on the mesiobuccal surfaces of the groups FG1 and FG30 according to the same protocol as in Recaldent tooth mousse groups^26^.

Then brackets from the first fluoride varnish group (FG1) and the first Recaldent group (RG1) were bonded and submitted to a tension test 1 day after application (T1). The teeth of the other two experimental groups (FG30 and RG30) were immersed in saline for 30 days (T2), changing it daily, and only then were brackets bonded and the teeth submitted to a tension test^19^.

Before the bonding procedure, the buccal surface of each tooth was polished with a rubber cup and non-fluoridated pumice, rinsed with water and air-dried. Following, the prepared enamel areas were etched with 37% phosphoric acid gel (i-GEL, i-dental Lietuva, Šiauliai, Lithuania) for 30 s, then washed and air-dried for 20 s until the surface appeared frosted. The etched buccal surface was coated with a thin layer of TruLock bond (Rocky Mountain Orthodontics, Denver, USA) and light-cured for 10 s (3 M ESPE Epilar, Neuss, Germany,1200 mW/cm2).

Directly afterwards, identical premolar metal braces (022 Roth, Discovery, Dentaurum, Ispringen, Germany) were bonded using light-cured TruLock adhesive resin (Rocky Mountain Orthodontics, Denver, USA). Each bracket was positioned 1 mm gingivally to the buccal cusp tip and pressed against the buccal tooth surface with an adapter using a force of 100 g (9.8 N), all done by the same person to ensure the standard thickness of the adhesive. A dental probe was used to remove residual adhesive around the bracket. Bracket adhesive was light-cured for 20 s (3 M ESPE Epilar, Neuss, Germany, 1200 mW/cm^2^)^27, 28^. All samples were kept in saline for 24 h after bonding to achieve complete resin polymerization.

**Figure 1:**
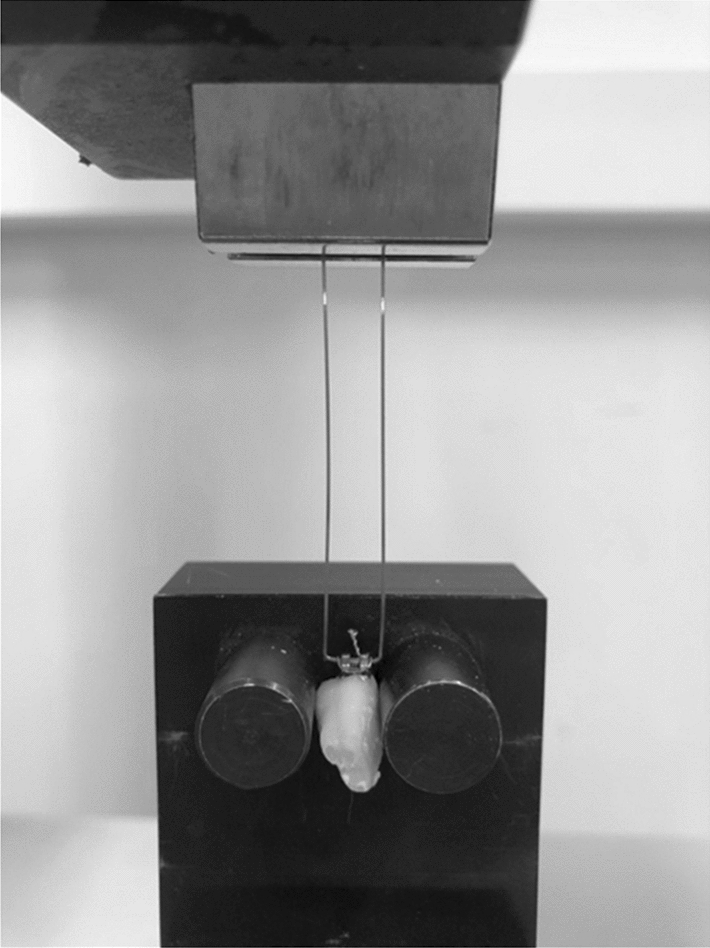
Tooth fixed in universal testing machine.

The tensile bond strength (TBS) was measured in the Department of Mechanical Engineering at the Kaunas University of Technology. The loops were bent from the orthodontic archwire and fixed to the brackets with ligatures (Fig. 1)^20^. Then they were adjusted to the universal mechanical testing machine (H24KT, Tinius Olsen, England). The testing machine was used at a crosshead speed of 5 mm/min until the bracket was debonded from the tooth. The highest debonding forces (N) of the brackets were recorded automatically by a digital software measurement system. The system consisted of a force sensor (SS50, Wagner Instruments, USA, 250 N × 0.1 N) and a controller with a display (BGI, Wagner Instruments, USA). TBS was calculated using the force’s value and the base of the bracket area value (1 MPa = 1 N/mm^2^)^20^.

After debonding the brackets, each bracket was analyzed with a stereomicroscope (Stemi 2000-CS, Zeiss, Oberkochen, Germany), and images were taken with a digital microscope camera (AxioCam, MRC 5, Zeiss, Oberkochen, Germany), which were superimposed on a 100 × 100 cell scale^20^. Adhesive residues were evaluated using the ARI index. The ARI values were divided into 6 groups according to the percentage of adhesive remnant on the bracket: 1—when there is no composite on the bracket base, 2—less than 20% of the composite remains on the bracket base, 3—composite residue covers 20–40% of the bracket base, 4—composite residue covers 41–60% of the bracket base; 5—composite residues cover 61–80% of the bracket base; 6—composite residues cover 81% or more of the bracket base^20, 27^.

Statistical analysis was performed using IBM SPSS 28.0.1.1. Tensile bond strength (TBS) data were analyzed by one-way ANOVA and Tukey posthoc methods of descriptive statistics. ARI data was analyzed by the Chi-Square method. The difference in results between the variables was considered statistically significant if *p* < 0.05.

## Results

### Tensile bond strength (TBS) analysis

The data obtained was normally distributed according to the Shapiro–Wilk test, so one-way ANOVA and Tukey post-hoc tests were used. Descriptive statistics, including mean, standard deviation, maximum and minimum adhesion force values for each group of brackets, are shown in Table 1. The analysis showed that a statistically significant (*p* < 0.001) difference in the means TBS was found between the fluoride group FG1 (5.47 MPa; SD = 0.91) when the brackets were debonded the day after application, and the control (7.10 MPa; SD = 0.81) and other experimental groups.

**Table 1:** Descriptive statistics of the groups and comparison of TBS values.

Group	n	Mean	SD	Min	Max
Control	80	7.10	0.81	5.74	9.17
FG1	20	5.47	0.91	3.14	7.03
RG1	20	6.80	0.86	5.19	8.89
FG30	20	6.66	0.82	5.53	8.32
RG30	20	7.05	0.69	5.87	8.10

n, sample size; SD, standard deviation; Min, the smallest value; Max, the biggest value; FG1, fluoride varnish group a day after application; FG30, fluoride varnish group 30 after application; RG1, Recaldent tooth mousse group a day after application, RG30, Recaldent tooth mousse group 30 days after application.

**Figure 2:**
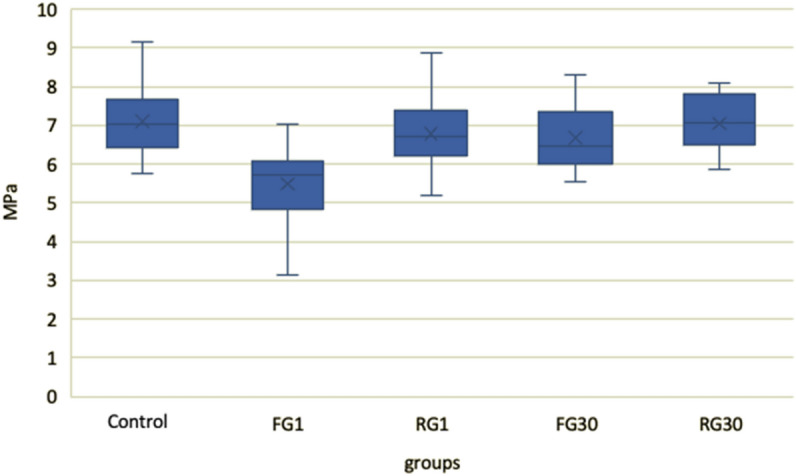
Average TBS of brackets compared by the different agents used. FG1—fluoride varnish group a day after application; FG30—fluoride varnish group 30 after application; RG1—Recaldent tooth mousse group a day after application, RG30—Recaldent tooth mousse group 30 days after application.

When the brackets were debonded 30 days after the application of fluoride varnish (6.66 MPa; SD = 0.82), the tensile bond strength was lower than in the control group, but no statistically significant difference was found (*p* = 0.215). No significant difference was found between Recaldent tooth mousse application and different debonding times compared to each other and the control group (*p* > 0.05) (Fig. 2.).

### ARI analysis

Table 2 shows the adhesive remnant index values. The Chi-Square test showed a statistically significantly higher amount of adhesive residue on the bracket of the FG1 group (*p* < 0.001) compared to the control group. No statistically significant difference in ARI was found between the other experimental groups and the control group.

**Table 2:** The distribution and frequency of the adhesive remnant index (ARI) between control and experimental groups.

Group	n	ARI						p* Value
		1	2	3	4	5	6	
Control	80	16 (20%)	20 (25%)	25 (31%)	12 (15%)	5 (6%)	2 (3%)	
FG1	20	0 (0%)	2 (10%)	3 (15%)	4 (20%)	7 (35%)	4 (20%)	< 0.001*
RG1	20	2 (10%)	4 (20%)	6 (30%)	6 (30%)	1 (5%)	1 (5%)	0.633
FG30	20	3 (15%)	4 (20%)	8 (40%)	3 (15%)	1 (5%)	1 (5%)	0.952
RG30	20	4 (20%)	5 (25%)	5 (25%)	4 (20%)	2 (10%)	0 (0%)	0.937

n, sample size; FG1, fluoride varnish group a day after application; FG30, fluoride varnish group 30 after application; RG1, Recaldent tooth mousse group a day after application, RG30, Recaldent tooth mousse group 30 days after application; *p < 0.05 (significant).

## Discussion

The development of caries before and during orthodontic treatment is a significant issue. Prevention of caries and orthodontic treatment are inevitable due to the high prevalence of caries and occlusal anomalies, which rank first and third in all oral diseases, respectively^16^. However, the influence on TBS of preventive measures of caries that include remineralizing agents is controversial.

In this study, the mean TBS for all groups fell within the Reynolds recommended range of optimal bracket adhesion equal to 5.9–7.8 MPa^18^ except for the fluoride varnish group, in which the brackets were bonded one day after fluoride varnish application (5.47 MPa; SD = 0.91).

Based on the results obtained and studies performed by other researchers, it can be assumed that the prophylactic use of fluoride leads to lower bracket retention. This is consistent with studies by Leódido et al., Daneshkazemi et al., Cossellu et al., in which the TBS ranged from 6.62 to 9.97 MPa in the fluoride group and from 12.82 to 17.38 MPa in the control group^19, 23, 26^. This may be since fluoride ions replace calcium ions in the surface layer of the enamel to form fluorapatites, which are more resistant to environmental influences, as well as phosphoric acid, which is used to etch the enamel. As a result, the depth of penetration of the bonding system into the enamel may decrease^26^. However, such a potential negative effect of fluoride on tensile bond strength should not outweigh its positive effects on prophylaxis.

When evaluating the effect of Recaldent tooth cream, the TBS was lower immediately after application of the cream (6.80 MPa; SD = 0.86) when compared to the control group (7.10 MPa; SD = 0.81) but this difference was not statistically significant (*p* > 0.05). Cehreli and co-authors as well as Dunne W.J. found that application of CPP-ACP prior to bonding reduced retention^28, 29^. Naseh et al.^30^ and Daneshkazemi et al.^26^ investigated that the use of CPP-ACP cream before fixation of the braces did increase the TBS compared to the control group, although not statistically significant. On the other hand, in studies by Xiaojun et al.^31^ and Kecik et al.^32^, this increase was statistically significant. Such different data is possible due to the different methods of application of the substances.

In this study, the application method of remineralizing agents was used as in the study by Daneshkazemi and co-authors^26^. Both Recaldent cream and fluoride varnish were applied to the tooth surface for 5 min, followed by rinsing with repeated water every 6 h for 5 days. The chosen methodology made it possible to simulate the in vivo study as much as possible but at the same time, to shorten the study time.

However, it is essential to note that in the study, the teeth were kept in saline but not in artificial saliva. According to Naseh and co-authors^30^, artificial saliva creates the most similar environment to oral conditions. However, in this study, the use of saline was chosen to determine the effect of remineralizing agents alone more objectively on tensile bond strength, ruling out the potential impact of salivary minerals.

Regarding ARI, this study found that a statistically significant amount of resin remained on the bracket in the fluoride varnish group when the brackets were bonded one day after the last application of the fluoride varnish, although in a study by Cossellu and co-authors^19^ no statistically significant difference was found between ARI values. No significant differences in ARI values between control and experimental groups were found in other studies as well^26, 30^. However, such results may have been affected by slightly different ARI assessment methodologies and score values^19, 21, 26, 30, 32^.

Finally, this study showed that remineralizing agents used to prevent caries have a few adverse effects on bracket adherence. That is especially pronounced in the first days after fluoride varnish applications, and the differences become insignificant after 30 days. GC Tooth Mousse Recaldent applications, meanwhile, have a significantly smaller impact. However, for even more accurate results, it would be appropriate to investigate an even larger sample in the future.

## Limitations of the study

This study was an in vitro study, thus it was impossible to repeat the tension test with the same teeth.

## Conclusion

Application of fluoride immediately before the bracket bonding procedure significantly reduced tensile bond strength, but after 30 days it was set back to an optimal value.

Application of fluoride immediately before the bracket bonding procedure resulted in the bond failure pattern and a significantly higher ARI score.

Application of CPP-ACP before the bracket bonding procedure had no adverse effect on tensile bond strength.

Application of CPP-ACP had no effect on the bond failure pattern and the ARI score.

## Data availability

A data can be available under reasonable request. If someone wants to request the data from this study a corresponding author Giedrė Trakinienė should be contacted; E-mail giedre.trakiniene@lsmuni.lt.
